# The Concise Guide to PHARMACOLOGY 2015/16: Overview

**DOI:** 10.1111/bph.13347

**Published:** 2015-12-09

**Authors:** Stephen PH Alexander, Eamonn Kelly, Neil Marrion, John A Peters, Helen E Benson, Elena Faccenda, Adam J Pawson, Joanna L Sharman, Christopher Southan, O Peter Buneman, William A Catterall, John A Cidlowski, Anthony P Davenport, Doriano Fabbro, Grace Fan, John C McGrath, Michael Spedding, Jamie A Davies, CGTP Collaborators, R Aldrich, B Attali, Ml Bäck, NM Barnes, R Bathgate, PM Beart, E Becirovic, M Biel, NJ Birdsall, D Boison, H Bräuner‐Osborne, S Bröer, C Bryant, G Burnstock, T Burris, D Cain, G Calo, SL Chan, KG Chandy, N Chiang, S Christakos, A Christopoulos, JJ Chun, J‐J Chung, DE Clapham, MA Connor, L Coons, HM Cox, FM Dautzenberg, G Dent, SD Douglas, ML Dubocovich, DP Edwards, R Farndale, TM Fong, D Forrest, CJ Fowler, P Fuller, RR Gainetdinov, MA Gershengorn, A Goldin, SAN Goldstein, SL Grimm, S Grissmer, AL Gundlach, B Hagenbuch, JR Hammond, JC Hancox, S Hartig, RL Hauger, DL Hay, T Hébert, AN Hollenberg, ND Holliday, D Hoyer, AP Ijzerman, KI Inui, S Ishii, KA Jacobson, LY Jan, GE Jarvis, R Jensen, A Jetten, R Jockers, LK Kaczmarek, Y Kanai, HS Kang, S Karnik, ID Kerr, KS Korach, CA Lange, D Larhammar, F Leeb‐Lundberg, R Leurs, SJ Lolait, D Macewan, JJ Maguire, JM May, J Mazella, CA Mcardle, DP Mcdonnell, MC Michel, LJ Miller, V Mitolo, T Monie, PN Monk, B Mouillac, PM Murphy, J‐L Nahon, J Nerbonne, CG Nichols, X Norel, R Oakley, S Offermanns, LG Palmer, MA Panaro, E Perez‐Reyes, RG Pertwee, JW Pike, JP Pin, S Pintor, LD Plant, DR Poyner, ER Prossnitz, S Pyne, D Ren, JK Richer, P Rondard, RA Ross, H Sackin, R Safi, MC Sanguinetti, CA Sartorius, DL Segaloff, FM Sladek, G Stewart, LA Stoddart, J Striessnig, RJ Summers, Y Takeda, M Tetel, L Toll, JS Trimmer, M‐J Tsai, SY Tsai, S Tucker, TB Usdin, J‐P Vilargada, M Vore, DT Ward, SG Waxman, P Webb, AD Wei, N Weigel, GB Willars, C Winrow, SS Wong, H Wulff, RD Ye, M Young, J‐M Zajac

**Affiliations:** ^1^ School of Biomedical Sciences University of Nottingham Medical School Nottingham NG7 2UH UK; ^2^ School of Physiology and Pharmacology University of Bristol Bristol BS8 1TD UK; ^3^ Neuroscience Division, Medical Education Institute, Ninewells Hospital and Medical School University of Dundee Dundee DD1 9SY UK; ^4^ Centre for Integrative Physiology University of Edinburgh Edinburgh EH8 9XD UK; ^5^ Laboratory for Foundations of Computer Science School of Informatics, University of Edinburgh Edinburgh EH8 9LE United Kingdom; ^6^ Department of Pharmacology University of Washington Seattle 98195‐7280 WA USA; ^7^ National Institute of Environmental Health Sciences, National Institutes of Health, Department of Health and Human Services, Research Triangle Park 27709 NC USA; ^8^ Clinical Pharmacology Unit University of Cambridge Cambridge CB2 0QQ UK; ^9^ PIQUR Therapeutics Basel 4057 Switzerland; ^10^ The Agnes Irwin School Rosemont Pennsylvania USA; ^11^ School of Life Sciences University of Glasgow Glasgow G12 8QQ UK; ^12^ Spedding Research Solutions SARL Le Vésinet 78110 France

## Abstract

The Concise Guide to PHARMACOLOGY 2015/16 provides concise overviews of the key properties of over 1750 human drug targets with their pharmacology, plus links to an open access knowledgebase of drug targets and their ligands (www.guidetopharmacology.org), which provides more detailed views of target and ligand properties. The full contents can be found at http://onlinelibrary.wiley.com/doi/10.1111/bph.13347/full. This compilation of the major pharmacological targets is divided into eight areas of focus: G protein‐coupled receptors, ligand‐gated ion channels, voltage‐gated ion channels, other ion channels, nuclear hormone receptors, catalytic receptors, enzymes and transporters. These are presented with nomenclature guidance and summary information on the best available pharmacological tools, alongside key references and suggestions for further reading. The Concise Guide is published in landscape format in order to facilitate comparison of related targets. It is a condensed version of material contemporary to late 2015, which is presented in greater detail and constantly updated on the website www.guidetopharmacology.org, superseding data presented in the previous Guides to Receptors & Channels and the Concise Guide to PHARMACOLOGY 2013/14. It is produced in conjunction with NC‐IUPHAR and provides the official IUPHAR classification and nomenclature for human drug targets, where appropriate. It consolidates information previously curated and displayed separately in IUPHAR‐DB and GRAC and provides a permanent, citable, point‐in‐time record that will survive database updates.

## Table of contents

**5729 Overview**

5734 Other Protein Targets

5734 Adiponectin receptors

5735 Blood coagulation components

5735 Non‐enzymatic BRD containing proteins

5736 Carrier proteins

5737 CD molecules

5738 Methyllysine reader proteins

5739 Cytokines and growth factors

5739 Fatty acid‐binding proteins

5741 Sigma receptors

5742 Tubulins

BPH13348::

**5744 G protein‐coupled receptors**

5746 Orphan and other 7TM receptors

5746 Class A Orphans

5756 Class C Orphans

5756Taste 1 receptors

5757 Taste 2 receptors

5758 Other 7TM proteins

5759 5‐Hydroxytryptamine receptors

5764 Acetylcholine receptors (muscarinic)

5766 Adenosine receptors

5768 Adhesion Class GPCRs

5770 Adrenoceptors

5774 Angiotensin receptors

5775 Apelin receptor

5777 Bile acid receptor

5778 Bombesin receptors

5780 Bradykinin receptors

5781 Calcitonin receptors

5783 Calcium‐sensing receptors

5784 Cannabinoid receptors

5785 Chemerin receptor

5785 Chemokine receptors

5791Cholecystokinin receptors

5792Class Frizzled GPCRs

5793 Complement peptide receptors

5795 Corticotropin‐releasing factor receptors

5796 Dopamine receptors

5798 Endothelin receptors

5799 G protein‐coupled estrogen receptor

5800 Formylpeptide receptors

5801 Free fatty acid receptors

5803 GABAB receptors

5805 Galanin receptors

5806 Ghrelin receptor

5807 Glucagon receptor family

5809 Glycoprotein hormone receptors

5810 Gonadotrophin‐releasing hormone receptors

5811 GPR18, GPR55 and GPR119

5812 Histamine receptors

5814 Hydroxycarboxylic acid receptors

5815 Kisspeptin receptor

5816 Leukotriene receptors

5818 Lysophospholipid (LPA) receptors

5819 Lysophospholipid (S1P) receptors

5820 Melanin‐concentrating hormone receptors

5821 Melanocortin receptors

5822 Melatonin receptors

5823 Metabotropic glutamate receptors

5826 Motilin receptor

5827 Neuromedin U receptors

5828 Neuropeptide FF/neuropeptide AF receptors

5829 Neuropeptide S receptor

5828 Neuropeptide W/neuropeptide B receptors

5830 Neuropeptide Y receptors

5832 Neurotensin receptors

5833 Opioid receptors

5835 Orexin receptors

5836 Oxoglutarate receptor

5836 P2Y receptors

5838 Parathyroid hormone receptors

5839 Platelet‐activating factor receptor

5840 Prokineticin receptors

5841 Prolactin‐releasing peptide receptor

5842 Prostanoid receptors

5844 Proteinase‐activated receptors

5844 QRFP receptor

5846 Relaxin family peptide receptors

5848 Somatostatin receptors

5850 Succinate receptor

5850 Tachykinin receptors

5852 Thyrotropin‐releasing hormone receptors

5852 Trace amine receptor

5854 Urotensin receptor

5854 Vasopressin and oxytocin receptors

5856 VIP and PACAP receptors

**5870 Ligand‐Gated Ion Channels**

5871 5‐HT3 receptors

5873 Acid‐sensing (proton‐gated) ion channels (ASICs)

5875 Epithelial sodium channels (ENaC)

5877 GABAA receptors

5882 Glycine receptors

5885 Ionotropic glutamate receptors

5891 IP3 receptor

5891 Nicotinic acetylcholine receptors

5896 P2X receptors

5898 Ryanodine receptor

5900 ZAC

**5904 Voltage‐gated ion channels**

5905 CatSper and Two‐Pore channels

5907 Cyclic nucleotide‐regulated channels

5909 Potassium channels

5910 Calcium‐activated potassium channels

5912 Inwardly rectifying potassium channels

5915 Two‐P potassium channels

5917 Voltage‐gated potassium channels

5920 Transient Receptor Potential channels

5934 Voltage‐gated calcium channels

5936 Voltage‐gated proton channel

5937 Voltage‐gated sodium channels

**5942 Other ion channels**

5943 Aquaporins

5944 Chloride channels

5944 ClC family

5947 CFTR

5948 Calcium activated chloride channel

5949 Maxi chloride channel

5950 Volume regulated chloride channels

5952 Connexins and Pannexins

5954 Sodium leak channel, non‐selective

**5956 Nuclear hormone receptors**

5958 1A. Thyroid hormone receptors

5959 1B. Retinoic acid receptors

5960 1C. Peroxisome proliferator‐activated receptors

5961 1D. Rev‐Erb receptors

5962 1F. Retinoic acid‐related orphans

5963 1H. Liver X receptor‐like receptors

5964 1I. Vitamin D receptor‐like receptors

5965 2A. Hepatocyte nuclear factor‐4 receptors

5966 2B. Retinoid X receptors

5967 2C. Testicular receptors

5968 2E. Tailless‐like receptors

5969 2F. COUP‐TF‐like receptors

5970 3B. Estrogen‐related receptors

5971 4A. Nerve growth factor IB‐like receptors

5972 5A. Fushi tarazu F1‐like receptors

5973 6A. Germ cell nuclear factor receptors

5974 0B. DAX‐like receptors

5975 Steroid hormone receptors

5975 3A. Estrogen receptors

5976 3C. 3‐Ketosteroid receptors

**5979 Catalytic receptors**

5981 Cytokine receptor family

5981 IL‐2 receptor family

5983 IL‐3 receptor family

5983 IL‐6 receptor family

5985 IL‐12 receptor family

5985 Prolactin receptor family

5986 Interferon receptor family

5987 IL‐10 receptor family

5988 Immunoglobulin‐like family of IL‐1 receptors

5889 IL‐17 receptor family

5890 GDNF receptor family

5891 Integrins

5994 Natriuretic peptide receptor family

5996 Pattern recognition receptors

5996 Toll‐like receptor family

5997 NOD‐like receptor family

5999 Receptor serine/threonine kinase (RSTK) family

6000 Type I receptor serine/threonine kinases

6001 Type II receptor serine/threonine kinases

6001 Type III receptor serine/threonine kinases

6002 RSTK functional heteromers

6003 Receptor tyrosine kinases

6004 Type I RTKs: ErbB (epidermal growth factor) receptor family

6005 Type II RTKs: Insulin receptor family

6005 Type III RTKs: PDGFR, CSFR, Kit, FLT3 receptor family

6007 Type IV RTKs: VEGF (vascular endothelial growth factor) receptor family

6008 Type V RTKs: FGF (fibroblast growth factor) receptor family

6008 Type VI RTKs: PTK7/CCK4

6009 Type VII RTKs: Neurotrophin receptor/Trk family

6010 Type VIII RTKs: ROR family

6010 Type IX RTKs: MuSK

6010 Type X RTKs: HGF (hepatocyte growth factor) receptor family

6011 Type XI RTKs: TAM (TYRO3‐, AXL‐ and MER‐TK) receptor family

6012 Type XII RTKs: TIE family of angiopoietin receptors

6012 Type XIII RTKs: Ephrin receptor family

6013 Type XIV RTKs: RET

6014 Type XV RTKs: RYK

6014 Type XVI RTKs: DDR (collagen receptor) family

6015 Type XVII RTKs: ROS receptors

6015 Type XVIII RTKs: LMR family

6016 Type XIX RTKs: Leukocyte tyrosine kinase (LTK) receptor family

6016 Type XX RTKs: STYK1

6017 Receptor tyrosine phosphatases (RTP)

6018 Tumour necrosis factor (TNF) receptor family

**6024 Enzymes**

6028 Protein Kinases (EC 2.7.x.x)

6028 Rho kinase

6029 Protein kinase C (PKC)

6029 Alpha subfamily

6029 Delta subfamily

6030 Eta subfamily

6030 FRAP subfamily

6031 CDK4 subfamily

6031 GSK subfamily

6032 Polo‐like kinase (PLK) family

6032 STE7 family

6033 Abl family

6033 Ack family

6034 Janus kinase (JakA) family

6034 Src family

6035 Tec family

6035 RAF family

6036 Peptidases and proteinases

6036 A1: Pepsin

6037 A22: Presenilin

6037 C14: Caspase

6037 M1: Aminopeptidase N

6038 M2: Angiotensin‐converting (ACE and ACE2)

6038 M10: Matrix metallopeptidase

6039 M12: Astacin/Adamalysin

6039 M28: Aminopeptidase Y

6040 M19: Membrane dipeptidase

6040 S1: Chymotrypsin

6041 T1: Proteasome

6042 S8: Subtilisin

6042 S9: Prolyl oligopeptidase

6042 Acetylcholine turnover

6044 Adenosine turnover

6045 Amino acid hydroxylases

6046 L‐Arginine turnover

6047 Arginase

6047 Arginine:glycine amidinotransferase

6047 Dimethylarginine dimethylaminohydrolases

6048 Nitric oxide synthases

6048 Carboxylases and decarboxylases

6049 Carboxylases

6050 Decarboxylases

6052 Catecholamine turnover

6055 Ceramide turnover

6055 Serine palmitoyltransferase

6056 Ceramide synthase

6057 Sphingolipid ??4‐desaturase

6058 Sphingomyelin synthase

6058 Sphingomyelin phosphodiesterase

6059 Neutral sphingomyelinase coupling factors

6059 Ceramide glucosyltransferase

6060 Acid ceramidase

6060 Neutral ceramidases

6061 Alkaline ceramidases

6061 Ceramide kinase

6062 Chromatin modifying enzymes

6062 2.1.1.‐ Protein arginine N‐methyltransferases

6062 3.5.1.‐ Histone deacetylases (HDACs)

6063 Cyclic nucleotide turnover

6063 Adenylyl cyclases

6064 Soluble guanylyl cyclase

6065 Exchange protein activated by cyclic AMP (Epac)

6066 Phosphodiesterases, 3',5'‐cyclic nucleotide

6069 Cytochrome P450

6069 CYP1 family

6070 CYP2 family

6070 CYP3 family

6071 CYP4 family

6072 CYP5, CYP7 and CYP8 families

6072 CYP11, CYP17, CYP19, CYP20 and CYP21 families

6073 CYP24, CYP26 and CYP27 families

6074 CYP39, CYP46 and CYP51 families

6075 Eicosanoid turnover

6076 Endocannabinoid turnover

6077 Cyclooxygenase

6077 Prostaglandin synthases

6079 Lipoxygenases

6080 Leukotriene and lipoxin metabolism

6081 GABA turnover

6082 Glycerophospholipid turnover

6082 Phosphatidylinositol kinases

6083 1‐phosphatidylinositol 4‐kinase family

6083 Phosphatidylinositol‐4‐phosphate 3‐kinase family

6084 Phosphatidylinositol 3‐kinase family

6084 Phosphatidylinositol‐4,5‐bisphosphate 3‐kinase family

6085 1‐phosphatidylinositol‐3‐phosphate 5‐kinase family

Type I PIP kinases (1‐phosphatidylinositol‐4‐phosphate 5‐kinase family)

6086 Type II PIP kinases (1‐phosphatidylinositol‐5‐phosphate 4‐kinase family)

6087 Phosphoinositide‐specific phospholipase C

6088 Phospholipase A_2_

6089 Phosphatidylcholine‐specific phospholipase D

6090 Lipid phosphate phosphatases

6091 Haem oxygenase

6092 Hydrogen sulphide synthesis

6093 Hydrolases

6093 Inositol phosphate turnover

6094 Inositol 1,4,5‐trisphosphate 3‐kinases

6094 Inositol polyphosphate phosphatases

6094 Inositol monophosphatase

6095 Lanosterol biosynthesis pathway

6097 Nucleoside synthesis and metabolism

6099 Sphingosine 1‐phosphate turnover

6100 Sphingosine kinase

6100 Sphingosine 1‐phosphate phosphatase

6101 Sphingosine 1‐phosphate lyase

6101 Thyroid hormone turnover

6103 1.14.11.29 2‐oxoglutarate oxygenases

6103 2.4.2.30 poly(ADP‐ribose)polymerases

6104 2.5.1.58 Protein farnesyltransferase

6104 3.5.3.15 Peptidyl arginine deiminases (PADI)

6104 RAS subfamily

6105 4.2.1.1 Carbonate dehydratases

6105 5.99.1.2 DNA Topoisomerases

**6110 Transporters**

6113 ATP‐binding cassette transporter family

6113 ABCA subfamily

6115 ABCB subfamily

6116 ABCC subfamily

6117 ABCD subfamily of peroxisomal ABC transporters

6118 ABCG subfamily

6119 F‐type and V‐type ATPases

6119 F‐type ATPase

6120 V‐type ATPase

6120 P‐type ATPases

6121 Na^+^/K^+^‐ATPases

6121 Ca^2^^+^‐ATPases

6122 H^+^/K^+^‐ATPases

6122 Cu^+^‐ATPases

6122 Phospholipid‐transporting ATPases

6123 Major facilitator superfamily (MFS) of transporters

6123 SLC superfamily of solute carriers

6124 SLC1 family of amino acid transporters

6124 Glutamate transporter subfamily

6126 Alanine/serine/cysteine transporter subfamily

6127 SLC2 family of hexose and sugar alcohol

6127 Class I transporters

6128 Class II transporters

6129 Proton‐coupled inositol transporter

6129 SLC3 and SLC7 families of heteromeric amino acid transporters (HATs)

6130 SLC3 family

6130 SLC7 family

6131 SLC4 family of bicarbonate transporters

6132 Anion exchangers

6132 Sodium‐dependent HCO_3_ transporters

6133 SLC5 family of sodium‐dependent glucose transporters

6134 Hexose transporter family

6135 Choline transporter

6136 Sodium iodide symporter, sodium‐dependent multivitamin transporter and sodium‐coupled monocarboxylate transporters

6137 Sodium *myo*‐inositol cotransporter transporters

6138 SLC6 neurotransmitter transporter family

6138 Monoamine transporter subfamily

6139 GABA transporter subfamily

6141 Glycine transporter subfamily

6142 Neutral amino acid transporter subfamily

6144 SLC8 family of sodium/calcium exchangers

6145 SLC9 family of sodium/hydrogen exchangers

6145 SLC10 family of sodium‐bile acid co‐transporters

6147 SLC11 family of proton‐coupled metal ion transporters

6148 SLC12 family of cation‐coupled chloride transporters

6149 SLC13 family of sodium‐dependent sulphate/carboxylate transporters

6150 SLC14 family of facilitative urea transporters

6151 SLC15 family of peptide transporters

6152 SLC16 family of monocarboxylate transporters

6154 SLC17 phosphate and organic anion transporter family

6154 Type I sodium‐phosphate co‐transporters

6155 Sialic acid transporter

6155 Vesicular glutamate transporters (VGLUTs)

6156 Vesicular nucleotide transporter

6156 SLC18 family of vesicular amine transporters

6158 SLC19 family of vitamin transporters

6159 SLC20 family of sodium‐dependent phosphate transporters

6160 SLC22 family of organic cation and anion transporters

6160 Organic cation transporters (OCT)

6161 Organic zwitterions/cation transporters (OCTN)

6162 Organic anion transporters (OATs)

6163 Urate transporter

6163 SLC23 family of ascorbic acid transporters

6164 SLC24 family of sodium/potassium/calcium exchangers

6165 SLC25 family of mitochondrial transporters

6165 Mitochondrial di‐ and tri‐carboxylic acid transporter subfamily

6166 Mitochondrial amino acid transporter subfamily

6167 Mitochondrial phosphate transporters

6167 Mitochondrial nucleotide transporter subfamily

6168 Mitochondrial uncoupling proteins

6169 Miscellaneous SLC25 mitochondrial transporters

6170 SLC26 family of anion exchangers

6170 Selective sulphate transporters

6171 Anion channels

6171 Other SLC26 anion exchangers

6172 SLC27 family of fatty acid transporters

6173 SLC28 and SLC29 families of nucleoside transporters

6173 SLC28 family

6174 SLC29 family

6176 SLC30 zinc transporter family

6176 SLC31 family of copper transporters

6177 SLC32 vesicular inhibitory amino acid transporter

6178 SLC33 acetylCoA transporter

6179 SLC34 family of sodium phosphate co‐transporters

6180 SLC35 family of nucleotide sugar transporters

6181 SLC36 family of proton‐coupled amino acid transporters

6182 SLC37 family of phosphosugar/phosphate exchangers

6182 SLC38 family of sodium‐dependent neutral amino acid transporters

6183 System A‐like transporters

6183 System N‐like transporters

6184 Orphan SLC38 transporters

6185 SLC39 family of metal ion transporters

6186 SLC40 iron transporter

6187 SLC41 family of divalent cation transporters

6187 SLC42 family of Rhesus glycoprotein ammonium transporters

6188 SLC43 family of large neutral amino acid transporters

6189 SLC44 choline transporter‐like family

6190 SLC45 family of putative sugar transporters

6191 SLC46 family of folate transporters

6192 SLC47 family ofmultidrug and toxin extrusion transporters

6192 SLC48 heme transporter

6193 SLC49 family of FLVCR‐related heme transporters

6194 SLC50 sugar transporter

6195 SLC51 family of steroid‐derived molecule transporters

6195 SLC52 family of riboflavin transporters

6196 SLCO family of organic anion transporting polypeptides

6199 Patched family

## Introduction

In order to allow clarity and consistency in pharmacology, there is a need for a comprehensive organisation and presentation of the targets of drugs. This is the philosophy of the IUPHAR/BPS Guide to PHARMACOLOGY presented on the online free access database (http://www.guidetopharmacology.org/). This database is supported by the British Pharmacological Society (BPS), the International Union of Basic and Clinical Pharmacology (IUPHAR), the Wellcome Trust and the University of Edinburgh. Data included in the Guide to PHARMACOLOGY are derived in large part from interactions with the subcommittees of the Nomenclature Committee of the International Union of Basic and Clinical Pharmacology (NC‐IUPHAR). The Editors of the Concise Guide have compiled the individual records, in concert with the team of Curators, drawing on the expert knowledge of these latter subcommittees. The tables allow an indication of the status of the nomenclature for the group of targets listed, usually previously published in Pharmacological Reviews. In the absence of an established subcommittee, advice from several prominent, independent experts has generally been obtained to produce an authoritative consensus on nomenclature, which attempts to fit in within the general guidelines from NC‐IUPHAR. This current edition, the Concise Guide to PHARMACOLOGY 2015/16, is the latest snapshot of the database in print form, following on from the Concise Guide to PHARMACOLOGY 2013/14. It contains data drawn from the online database as a rapid overview of the major pharmacological targets. Thus, there are fewer targets presented in the Concise Guide (1761) compared to the online database (2761, as of August 2015). The priority for inclusion in the Concise Guide is the presence of quantitative pharmacological data. This means that often orphan family members are not presented in the Concise Guide, although structural information is available on the online database. An expansion in the current version of the Concise Guide is the increased inclusion of approved drugs, which reflects the aim of the online database to reflect the clinical exploitation of human molecular targets. Although many of these agents are much less selective than the tool compounds listed to define individual targets or groups of targets, we have included them for the significant interest associated with their use and mechanisms of action. The emphasis on approved drugs means that the online database has been expanded to include 8024 ligands (as of August 2015), meaning that additional records now appear in the Concise Guide, primarily in the enzymes section. The organisation of the data is tabular (where appropriate) with a standardised format, where possible on a single page, intended to aid understanding of and comparison within a particular target group. The Concise Guide is intended as an initial resource, with links to additional reviews and resources for greater depth and information. Pharmacological and structural data focus primarily on human gene products, wherever possible, with links to HGNC gene nomenclature and UniProt IDs. In a few cases, where data from human proteins are limited, data from other species are indicated. Pharmacological tools listed are prioritised on the basis of selectivity and availability. That is, agents (agonists, antagonists, inhibitors, activators, etc.) are included where they are both available (by donation or from commercial sources, now or in the near future) AND the most selective. This edition of the Concise Guide is divided into nine sections, which comprise pharmacological targets of similar structure/function. These are G protein‐coupled receptors, ligand‐gated ion channels, voltage‐gated ion channels, other ion channels, catalytic receptors, nuclear hormone receptors, enzymes, transporters and other protein targets. A new aspect of the Concise Guide 2015/16 is that each of these sections contains a complete listing of the families available for inspection on the online database, identifying those families reported in the Concise Guide by their page numbers. We hope that the Concise Guide will provide for researchers, teachers and students a state‐of‐the‐art source of accurate, curated information on the background to their work that they will use in the Introductions to their Research Papers or Reviews, or in supporting their teaching and studies.

We recommend that any citations to information in the Concise Guide are presented in the following format:

Alexander SPH et al. (2015). The Concise Guide to PHARMACOLOGY 2015/16: Overview. Br J Pharmacol XXX.

In this overview are listed protein targets of pharmacological interest, which are not G protein‐coupled receptors, ligand‐gated ion channels, voltage‐gated ion channels, ion channels, nuclear hormone receptors, catalytic receptors, transporters or enzymes.

## A dedication

This Edition of the Concise Guide to PHARMACOLOGY is dedicated to Tony Harmar (1951‐2014). Tony was a friend and colleague, who was involved with IUPHAR for over 15 years and worked on the IUPHAR database for over a decade at Edinburgh, working hard to establish the curators as a team of highly informed and informative individuals imbued with Tony's passion and dogged determination to focus on high‐quality data input, ensuring high‐quality data output. With time and the resources of the BPS and Wellcome Trust, combined with the expertise of the NC‐IUPHAR committee members mentioned above, Tony established the online database at http://www.guidetopharmacology.org/ as the exceptional resource it is today.

## Acknowledgements

We are extremely grateful for the financial contributions from the British Pharmacological Society, the International Union of Basic and Clinical Pharmacology, the Wellcome Trust (099156/Z/12/Z]), which support the website and the University of Edinburgh, who host the guidetopharmacology.org website. We are also tremendously grateful to the long list of collaborators from NC‐IUPHAR subcommittees and beyond, who have assisted in the construction of the Concise Guide to PHARMACOLOGY 2015/16 and the online database www.GuideToPHARMACOLOGY.org.

## Conflict of interest

The authors state that there are no conflicts of interest to disclose.

## Other Protein Targets

### Family structure

5734 Adiponectin receptors

‐ B‐cell lymphoma 2 (Bcl‐2) protein family

5735 Blood coagulation components

‐ Bromodomain‐containing proteins

5735 Non‐enzymatic BRD containing proteins

5736 Carrier proteins

5737 CD molecules

‐ Chromatin‐interacting transcriptional repressors

5738 Methyllysine reader proteins

‐ Circadian clock proteins

5739 Cytokines and growth factors

‐ EF‐hand domain containing

5739 Fatty acid‐binding proteins

‐ Heat shock proteins

‐ Immunoglobulins

‐ Inhibitors of apoptosis (IAP) protein family

‐ Kelch‐like proteins

‐ Kinesins

‐ Mitochondrial‐associated proteins

‐ Notch receptors

‐ Pentaxins

‐ Serum pentaxins

‐ Regulators of G protein signaling (RGS) proteins

‐ RZ family

‐ R4 family

‐ R7 family

‐ R12 family

‐ Reticulons

‐ Ribosomal factors

5741 Sigma receptors

5742 Tubulins

‐ Tumour‐associated proteins

‐ WD repeat‐containing proteins

## 1 Adiponectin receptors

### 1.1 Overview

Adiponectin receptors (**provisional nomenclature**, ENSFM00500000270960) respond to the 30 kDa complement‐related protein hormone adiponectin (also known as *ADIPOQ*: adipocyte, C1q and collagen domain‐containing protein; ACRP30, adipose most abundant gene transcript 1; apM‐1; gelatin‐binding protein: Q15848) originally cloned from adipocytes [49]. Although sequence data suggest 7TM domains, immunological evidence indicates that, contrary to typical 7TM topology, the carboxyl terminus is extracellular, while the amino terminus is intracellular [86]. Signalling through these receptors appears to avoid G proteins. Adiponectin receptors appear rather to stimulate protein phosphorylation via AMP‐activated protein kinase and MAP kinase pathways [86], possibly through the protein partner *APPL1* (adaptor protein, phosphotyrosine interaction, PH domain and leucine zipper containing 1, Q9UKG1[52]). The adiponectin receptors are a class of proteins (along with membrane progestin receptors), which contain seven sequences of aliphatic amino acids reminiscent of GPCRs, but which are structurally and functionally distinct from that class of receptor.

**Table:** 

Nomenclature	Adipo1 receptor	Adipo2 receptor
HGNC, UniProt	*ADIPOR1*, Q96A54	*ADIPOR2*, Q86V24
Rank order of potency	globular adiponectin (*ADIPOQ*, Q15848) >adiponectin (*ADIPOQ*, Q15848)	globular adiponectin (*ADIPOQ*, Q15848) = adiponectin (*ADIPOQ*, Q15848)

### 1.2 Comments

T‐Cadherin (*CDH13*, P55290) has also been suggested to be a receptor for (hexameric) adiponectin [35].

### 1.3 Further Reading

Buechler C *et al*. (2010) Adiponectin receptor binding proteins–recent advances in elucidating adiponectin signalling pathways. *FEBS Lett.* **584**: 4280‐6 [PMID:20875820]

Dalamaga M *et al*. (2012) The role of adiponectin in cancer: a review of current evidence. *Endocr. Rev.* **33**: 547‐94 [PMID:22547160]

Goldstein BJ *et al*. (2009) Protective vascular and myocardial effects of adiponectin. *Nat Clin Pract Cardiovasc Med* **6**: 27‐35 [PMID:19029992]

Juhl C *et al*. (2012) Molecular tools to characterize adiponectin activity. *Vitam. Horm.* **90**: 31‐56 [PMID:23017711]

Shetty S *et al*. (2009) Adiponectin in health and disease: evaluation of adiponectin‐targeted drug development strategies. *Trends Pharmacol. Sci.* **30**: 234‐9 [PMID:19359049]

Sun Y *et al*. (2009) Adiponectin, an unlocking adipocytokine. *Cardiovasc Ther* **27**: 59‐75 [PMID:19207481]

Thundyil J *et al*. (2012) Adiponectin receptor signalling in the brain. *Br. J. Pharmacol.* **165**: 313‐27 [PMID:21718299]

## 2 Blood coagulation components

### 2.1 Overview

Coagulation as a patho/physiological process is interpreted as a mechanism for reducing excessive blood loss through the generation of a gel‐like clot local to the site of injury. The process involves the activation, adhesion (see Integrins), degranulation and aggregation of platelets, as well as proteins circulating in the plasma. The coagulation cascade involves multiple proteins being converted to more active forms from less active precursors, typically through proteolysis (see Proteases). Listed here are the components of the coagulation cascade targetted by agents in current clinical usage.

**Table:** 

Nomenclature	coagulation factor V (proaccelerin, labile factor)	coagulation factor VIII, procoagulant component	serpin peptidase inhibitor, clade C (antithrombin), member 1
HGNC, UniProt	*F5*, P12259	*F8*, P00451	*SERPINC1*, P01008
Selective activators	–	–	heparin (p*K*_d_ 7.8) [25], fondaparinux (p*K*_d_ 7.5) [65], dalteparin [34], danaparoid [15, 58], enoxaparin [17], tinzaparin [19]
Selective antagonists	drotrecogin alfa (Inhibition) [40, 41]	drotrecogin alfa (Inhibition) [40, 41]	–

### 2.2 Further Reading

Astermark J (2015) FVIII inhibitors: pathogenesis and avoidance. *Blood* **125**: 2045‐2051 blue[PMID:25712994]

## 3 Non‐enzymatic BRD containing proteins

### 3.1 Overview

bromodomains bind proteins with acetylated lysine residues, such as histones, to regulate gene transcription. Listed herein are examples of bromodomain‐containing proteins for which sufficient pharmacology exists.

**Table:** 

Nomenclature	bromodomain adjacent to zinc finger domain, 2A	bromodomain adjacent to zinc finger domain, 2B	CREB binding protein	polybromo 1	SWI/SNF related, matrix associated, actin dependent regulator of chromatin, subfamily a, member 4
HGNC, UniProt	*BAZ2A*, Q9UIF9	*BAZ2B*, Q9UIF8	*CREBBP*, Q92793	*PBRM1*, Q86U86	*SMARCA4*, P51532
Selective inhibitors	GSK2801 (p*K*_d_ 6.6) 73	GSK2801 (Binding) (p*K*_d_ 6.9) 73	I‐CBP112 (p*K*_d_ 6.8) 72	PFI‐3 (Binding) (p*K*_d_ 7.3) 79	PFI‐3 (Binding) (p*K*_d_ 7.1) 79

### 3.2 Further Reading

Brand M *et al*. (2015) Small molecule inhibitors of bromodomain‐acetyl‐lysine interactions. *ACS Chem Biol* **10**:22‐39 blue[PMID:25549280]

Filippakopoulos P and Knapp S (2014) Targeting bromodomains: epigenetic readers of lysine acetylation. *Nat Rev Drug Discov* **13**: 337‐356 blue[PMID:24751816]

Gallenkamp D *et al*. (2014) Bromodomains and their pharmacological inhibitors. *ChemMedChem* **9**: 438‐464 blue[PMID:24497428]

Sanchez R *et al*. (2014) The bromodomain: from epigenome reader to druggable target. *Biochim Biophys Acta* **1839**: 676‐685 blue[PMID:24686119]

## 4 Carrier proteins

### 4.1 Overview

TTR is a homo‐tetrameric protein which transports thyroxine in the plasma and cerebrospinal fluid and retinol (vitamin A) in the plasma. Many disease causing mutations in the protein have been reported, many of which cause complex dissociation and protein mis‐assembly and deposition of toxic aggregates amyloid fibril formation [66]. These amyloidogenic mutants are linked to the development of pathological amyloidoses, including familial amyloid polyneuropathy (FAP) [1, 13], familial amyloid cardiomyopathy (FAC) [37], amyloidotic vitreous opacities, carpal tunnel syndrome [57] and others. In old age, non‐mutated TTR can also form pathological amyloid fibrils [85]. Pharmacological intervention to reduce or prevent TTR dissociation is being pursued as a theapeutic strategy. To date one small molecule kinetic stabilising molecule (tafamidis) has been approved for FAP, and is being evaluated in clinical trials for other TTR amyloidoses.

**Table:** 

Nomenclature	transthyretin
Common abreviation	TTR
HGNC, UniProt	*TTR*, P02766

## 5 CD molecules

### 5.1 Overview

Cluster of differentiation refers to an attempt to catalogue systematically a series of over 300 cell‐surface proteins associated with immunotyping. Many members of the group have identified functions as enzymes (for example, see CD73 ecto‐5'‐nucleotidase) or receptors (for example, see CD41 integrin, alpha 2b subunit). Many CDs are targetted for therapeutic gain using antibodies for the treatment of proliferative disorders. A full listing of all the Clusters of Differentiation is not possible in the Guide to PHARMACOLOGY; listed herein are selected members of the family targetted for therapeutic gain.

**Table:** 

Nomenclature	CD2	CD3e molecule, epsilon (CD3‐TCR complex)	CD20 (membrane‐spanning 4‐domains, subfamily A, member 1)	CD33	CD52	CD80	CD86	cytotoxic T‐lymphocyte‐associated protein 4 (CD152)
Common abreviation	–	–	–	–	–	–	–	CTLA‐4
HGNC, UniProt	*CD2*, P06729	*CD3E*, P07766	*MS4A1*, P11836	*CD33*, P20138	*CD52*, P31358	*CD80*, P33681	*CD86*, P42081	*CTLA4*, P16410
Selective inhibitors	–	–	–	–	–	abatacept [84], belatacept [16]	abatacept [84], belatacept [16]	–
Selective antagonists	alefacept (Inhibition) [56, 89]	–	–	–	–	–	–	–
Antibodies	–	catumaxomab (Binding) [46], muromonab‐CD3 (Binding) [24], otelixizumab (Binding) [7]	ofatumumab (Binding) (p*K*_d_ 9.9) 47, rituximab (Binding) (p*K*_d_ 8.5) [78], ibritumomab tiuxetan (Binding), obinutuzumab (Binding) [2, 68], tositumomab (Binding)	lintuzumab (Binding) (p*K*_d_∼10) [8], gemtuzumab ozogamicin (Binding) [6]	alemtuzumab (Binding) [22]	–	–	ipilimumab (Binding) (p*K*_d_>9) 28, tremelimumab (Binding) (p*K*_d_ 8.9) 30

**Table:** 

Nomenclature	programmed cell death 1 (CD279)
Common abreviation	PD‐1
HGNC, UniProt	*PDCD1*, Q15116
Antibodies	pembrolizumab (Binding) (p*K*_d_∼10) 9, nivolumab (Binding) (p*K*_d_ 9.1) [29, 42, 43]
Comments	The endogenous ligands for human PD‐1 are programmed cell death 1 ligand 1 (PD‐L1 *aka* CD274 (*CD274*, Q9NZQ7)) and programmed cell death 1 ligand 2 (PD‐L2; *PDCD1LG2*). These ligands are cell surface peptides, normally involved in immune system regulation. Many types of cancer cells evolve mechanisms to evade control and elimination by the immune system. Such mechanisms can include inhibition of so‐called 'immune checkpoints', which would normally be involved in the maintenance of immune homeostasis. An increasingly important area of clinical oncology research is the development of new agents which impede these evasion techniques, thereby switching immune vigilance back on, and effecting immune destruction of cancer cells. Three molecular targets of checkpoint inhibitors which are being extensively pursued are cytotoxic T‐lymphocyte antigen 4 (CTLA4), programmed cell death 1 (PD‐1), and programmed cell death ligand 1 (PD‐L1). Using antibody‐based therapies targeting these pathways, clinical responses have been reported in various tumour types, including melanoma, renal cell carcinoma [64] and non‐small cell lung cancer [39, 51]. pembrolizumab is the first‐in‐class, anti‐PD‐1 antibody to be approved by the US FDA, with ongoing clinical trials for nivolumab (*e.g.* NCT01673867, NCT01721746) and pidilizumab (NCT02077959, NCT01952769).

## 6 Methyllysine reader proteins

### 6.1 Overview

Methyllysine reader proteins bind to methylated proteins, such as histones, allowing regulation of gene expression.

**Table:** 

Nomenclature	l(3)mbt‐like 3 (Drosophila)
HGNC, UniProt	*L3MBTL3*, Q96JM7
Selective agonists	UNC1215 (p*K*_d_ 6.9) [38]

### 6.2 Further Reading

Liu K *et al*. (2015) Epigenetic targets and drug discovery Part 2: Histone demethylation and DNA methylation. *Pharmacol Ther* **151**: 121‐140 blue[PMID:25857453]

Musselman CA *et al*. (2014) Towards understanding methyllysine readout. *Biochim Biophys Acta* **1839**: 686‐693 blue[PMID:24727128]

Thinnes CC *et al*. (2014) Targeting histone lysine demethylases ‐ progress, challenges, and the future. *Biochim Biophys Acta* **1839**: 1416‐1432 blue[PMID:24859458]

## 7 Cytokines and growth factors

### 7.1 Overview

cytokines and growth factors are a group of small proteins released from cells, which act upon the same cell or neighbouring cells, often with a role in immune regulation and/or proliferation. Listed herein are examples of cytokines and growth factors targetted for therapeutic benefit.

**Table:** 

Nomenclature	interleukin 1, beta	tumor necrosis factor	vascular endothelial growth factor A
HGNC, UniProt	*IL1B*, P01584	*TNF*, P01375	*VEGFA*, P15692
Antagonists	–	–	aflibercept (Inhibition) [10, 11, 82]
Selective antagonists	–	etanercept (Inhibition) [18, 23]	pegaptanib (Inhibition) [26, 61]
Antibodies	gevokizumab (Binding) (p*K*_d_ 12.5) [36, 53, 71], canakinumab (Binding) (p*K*_d_ 10.5) 27, rilonacept (Binding) [32, 55]	golimumab (Inhibition) (pIC_50_ 10.7) [77], infliximab (Inhibition) (p*K*_d_ 8.7) 44, adalimumab (Inhibition) (p*K*_d_>8) 75, certolizumab pegol (Inhibition) [60]	ranibizumab (Inhibition) (p*K*_d_∼9.8) 3, bevacizumab (Inhibition) (pIC_50_ 8–8.3) 3

## 8 Fatty acid‐binding proteins

### 8.1 Overview

Fatty acid‐binding proteins are low molecular weight (100‐130 aa) chaperones for long chain fatty acids, fatty acyl CoA esters, eicosanoids, retinols, retinoic acids and related metabolites and are usually regarded as being responsible for allowing the otherwise hydrophobic ligands to be mobile in aqueous media. These binding proteins may perform functions extracellularly (*e.g.* in plasma) or transport these agents; to the nucleus to interact with nuclear receptors (principally PPARs and retinoic acid receptors [76]) or for interaction with metabolic enzymes. Although sequence homology is limited, crystallographic studies suggest conserved 3D structures across the group of binding proteins.

**Table:** 

Nomenclature	fatty acid binding protein 1, liver	fatty acid binding protein 2, intestinal	fatty acid binding protein 3, muscle and heart	fatty acid binding protein 4, adipocyte	fatty acid binding protein 5 (psoriasis‐associated)
HGNC, UniProt	*FABP1*, P07148	*FABP2*, P12104	*FABP3*, P05413	*FABP4*, P15090	*FABP5*, Q01469
Rank order of potency	stearic acid, oleic acid>palmitic acid, linoleic acid>arachidonic acid, *α*‐linolenic acid [69]	stearic acid>palmitic acid,oleic acid>linoleic acid>arachidonic acid, *α*‐linolenic acid [69]	stearic acid, oleic acid, palmitic acid>linoleic acid, *α*‐linolenic acid, arachidonic acid [69]	oleic acid, palmitic acid, stearic acid, linoleic acid>*α*‐linolenic acid, arachidonic acid [69]	–
Comments	A broader substrate specificity than other FABPs, binding two fatty acids per protein [83].	Crystal structure of the rat FABP2 [74].	Crystal structure of the human FABP3 [87].	–	Crystal structure of the human FABP5 [33].

**Table:** 

Nomenclature	fatty acid binding protein 6, ileal	fatty acid binding protein 7, brain	peripheral myelin protein 2	fatty acid binding protein 9, testis	fatty acid binding protein 12
HGNC, UniProt	*FABP6*, P51161	*FABP7*, O15540	*PMP2*, P02689	*FABP9*, Q0Z7S8	*FABP12*, A6NFH5
Comments	Able to transport bile acids [88].	Crystal structure of the human FABP7 [4].	*In silico* modelling suggests that FABP8 can bind both fatty acids and cholesterol [50].	–	–

**Table:** 

Nomenclature	retinol binding protein 1, cellular	retinol binding protein 2, cellular	retinol binding protein 3, interstitial	retinol binding protein 4, plasma	retinol binding protein 5, cellular
HGNC, UniProt	*RBP1*, P09455	*RBP2*, P50120	*RBP3*, P10745	*RBP4*, P02753	*RBP5*, P82980
Rank order of potency	–	stearic acid>palmitic acid, oleic acid, linoleic acid, *α*‐linolenic acid, arachidonic acid [70]	–	–	–

**Table:** 

Nomenclature	retinol binding protein 7, cellular	retinaldehyde binding protein 1	cellular retinoic acid binding protein 1	cellular retinoic acid binding protein 2
HGNC, UniProt	*RBP7*, Q96R05	*RLBP1*, P12271	*CRABP1*, P29762	*CRABP2*, P29373
Rank order of potency	–	11‐*cis*‐retinal, 11‐*cis*‐retinol>9‐*cis*‐retinal, 13‐*cis*‐retinal, 13‐*cis*‐retinol, all‐*trans*‐retinal, retinol [14]	tretinoin >alitretinoin stearic acid>palmitic acid, oleic acid, linoleic acid, *α*‐linolenic acid, arachidonic acid [70]	–

### 8.2 Comments

Although not tested at all FABPs, BMS309403 exhibits high affinity for FABP4 (pIC50  8.8) compared to FABP3 or FABP5 (pIC50 <6.6) [20, 81]. HTS01037 is reported to interfere with FABP4 action [31]. Multiple pseudogenes for the FABPs have been identified in the human genome.

### 8.3 Further Reading

Chmurzyńska A. (2006) The multigene family of fatty acid‐binding proteins (FABPs): function, structure and polymorphism. *J. Appl. Genet.* **47**: 39‐48 [PMID:16424607]

Furuhashi M *et al*. (2008) Fatty acid‐binding proteins: role in metabolic diseases and potential as drug targets. *Nat Rev Drug Discov* **7**: 489‐503 [PMID:18511927]

Kralisch S *et al*. (2013) Adipocyte fatty acid binding protein: a novel adipokine involved in the pathogenesis of metabolic and vascular disease? *Diabetologia* **56**: 10‐21 [PMID:23052058]

Schroeder F *et al*. (2008) Role of fatty acid binding proteins and long chain fatty acids in modulating nuclear receptors and gene transcription. *Lipids* **43**: 1‐17 [PMID:17882463]

Storch J *et al*. (2010) Tissue‐specific functions in the fatty acid‐binding protein family. *J. Biol. Chem.* **285**: 32679‐83 [PMID:20716527]

Yamamoto T *et al*. (2009) Classification of FABP isoforms and tissues based on quantitative evaluation of transcript levels of these isoforms in various rat tissues. *Biotechnol. Lett.* **31**: 1695‐701 [PMID:19565192]

## 9 Sigma receptors

### 9.1 Overview

Although termed ‘receptors’, the evidence for coupling through conventional signalling pathways is lacking. Initially described as a subtype of opioid receptors, there is only a modest pharmacological overlap and no structural convergence with the G protein‐coupled receptors. A wide range of compounds, ranging from psychoactive agents to antihistamines, have been observed to bind to these sites, which appear to be intracellular.

**Table:** 

Nomenclature	sigma non‐opioid intracellular receptor 1	*σ*2
HGNC, UniProt	*SIGMAR1*, Q99720	–
Agonists	–	PB‐28 (p*K*_i_ 8.3) [5], 1,3‐ditolylguanidine (p*K*_i_ 7.4) [45] – Guinea pig
(Sub)family‐selective agonists	(RS)‐PPCC (p*K*_i_ 8.8) [67]	–
Selective agonists	PRE‐084 (pIC_50_ 7.4) [80], (+)‐SK&F10047	–
Antagonists	(‐)‐pentazocine	SM 21 (pIC_50_ 7.2) [48]
Selective antagonists	NE‐100 (pIC_50_ 8.4) [62], BD‐1047 (pIC_50_ 7.4) [54]	–
Labelled ligands	[^3^H]pentazocine (Agonist)	[^3^H]‐di‐o‐tolylguanidine (Agonist)
Comments	–	There is no molecular correlate of the *σ*2 receptor.

### 9.2 Comments

### 9.3 Further Reading

Dubrovsky B. (2006) Neurosteroids, neuroactive steroids, and symptoms of affective disorders. *Pharmacol. Biochem. Behav.* **84**: 644‐55 [PMID:16962651]

Guitart X *et al*. (2004) Sigma receptors: biology and therapeutic potential. *Psychopharmacology (Berl.)* **174**: 301‐19 [PMID:15197533]

Matsumoto RR *et al*. (2003) Sigma receptors: potential medications development target for anti‐cocaine agents. *Eur. J. Pharmacol.* **469**: 1‐12 [PMID:12782179]

de Medina P *et al*. (2011) Importance of cholesterol and oxysterols metabolism in the pharmacology of tamoxifen and other AEBS ligands. *Chem. Phys. Lipids* **164**: 432‐7 [PMID:21641337]

## 10 Tubulins

### 10.1 Overview

Tubulins are a family of intracellular proteins most commonly associated with microtubules, part of the cytoskeleton. They are exploited for therapeutic gain in cancer chemotherapy as targets for agents derived from a variety of natural products: taxanes, colchicine and vinca alkaloids. These are thought to act primarily through *β*‐tubulin, thereby interfering with the normal processes of tubulin polymer formation and disassembly.

**Table:** 

Nomenclature	tubulin, alpha 1a	tubulin, alpha 4a	tubulin, beta class I	tubulin, beta 3 class III	tubulin, beta 4B class IVb	tubulin, beta 8 class VIII
HGNC, UniProt	*TUBA1A*, Q71U36	*TUBA4A*, P68366	*TUBB*, P07437	*TUBB3*, Q13509	*TUBB4B*, P68371	*TUBB8*, Q3ZCM7
Inhibitors	–	–	vinblastine (pIC_50_ 9), vincristine	–	–	–
(Sub)family‐selective inhibitors	–	–	eribulin (pIC_50_ 8.2) [59], paclitaxel (Mitotic cell cycle arrest in A431 cells) (pEC_50_ 8.1) [63], colchicine (pIC_50_ 8) [12], cabazitaxel, docetaxel, ixabepilone	–	–	–

### 10.2 Further Reading

Kaur R *et al*. (2014) Recent developments in tubulin polymerization inhibitors: An overview. *Eur J Med Chem* **87**: 89‐124 [PMID:25240869]

Lu Y *et al*. (2012) An overview of tubulin inhibitors that interact with the colchicine binding site. *Pharm. Res.* **29**: 2943‐71 [PMID:22814904]

Perdiz D *et al*. (2011) The ins and outs of tubulin acetylation: more than just a post‐translational modification? *Cell. Signal.* **23**: 763‐71 [PMID:20940043]

Schappi JM *et al*. (2014) Tubulin, actin and heterotrimeric G proteins: coordination of signaling and structure. *Biochim. Biophys. Acta* **1838**: 674‐81 [PMID:24071592]

Song Y *et al*. (2015) Post‐translational modifications of tubulin: pathways to functional diversity of microtubules. *Trends Cell Biol.* **25**: 125‐36 [PMID:25468068]

Yu I *et al*. (2015) Writing and Reading the Tubulin Code. *J. Biol. Chem.* **290**: 17163‐72 [PMID:25957412]
